# In-vivo effects of flapless osteopuncture-facilitated tooth movement in the maxilla and the mandible

**DOI:** 10.4317/jced.54981

**Published:** 2018-08-01

**Authors:** Tugba Haliloglu-Ozkan, Nursel Arici, Selim Arici

**Affiliations:** 1 Phd, DDS, Research Assistant, Department of Orthodontics, Faculty of Dentistry, Ondokuz Mayıs University, Samsun, Turkey; 2 Associate Professor, Department of Orthodontics, Faculty of Dentistry, Ondokuz Mayıs University, Samsun, Turkey; 3 Professor, Department of Orthodontics, Faculty of Dentistry, Ondokuz Mayıs University Samsun, Turkey

## Abstract

**Background:**

This study aimed to investigate the effects of a minimally invasive, flapless procedure to enhance tooth movement in both jaws and to determine whether this triggers the acceleration when repeated monthly.

**Material and Methods:**

The sample consisted of thirty-two individuals whose orthodontic treatment required canine retraction. They were divided into an experimental group and control group. Osteopunctures were performed using orthodontic mini-screws at the distal aspects of the canine teeth at the beginning and on the fourth week of distalization in the experimental group. The control group was treated with conventional mechanics. All canines were retracted. The rates of canine distalization, rotation, and tipping were measured on the first, fourth, and eighth weeks of distalization. First molar anchorage loss was also measured. Intergroup and intragroup analyses were performed.

**Results:**

Flapless osteopuncture-facilitated tooth movement resulted in greater canine distalization and reduced molar movement in the maxilla in the experimental than in the control group during the first month of distalization. In addition, the extent of upper canine movement was significantly higher in the experimental group in the first month than in the second. No differences in canine and molar movement in the mandible were observed between the two groups.

**Conclusions:**

OP, as applied in this study, is an effective method for increasing the rate of tooth movement in the maxilla. Repeating the procedure monthly does not appear to show a major advance of tooth movement.

** Key words:**Accelerated tooth movement, flapless osteopuncture.

## Introduction

The growing awareness of good health has increased the demand for orthodontic treatment. The duration of orthodontic treatment is the most important factor negatively affecting the treatment process. Prolonged treatment not only affects the psychosocial state of the patient but also increases the risk of periodontal disease, tooth decay, and root resorption (1). Therefore, researchers have attempted to identify various methods to enhance the rate of tooth movement.

Surgical procedures to alleviate alveolar integrity and accelerate OTM have been tested for almost 100 years. Traditional selective alveolar decortication methods (2,3) required invasive surgical insult to the bone, periosteum, and mucosa, which is unpleasant for the patient. In recent years, the traditional methods have been replaced by minimally invasive techniques involving, for example, piezoelectricity (4), corticision (5), and osteoperforations (6). Approaches to reduce osseos and soft tissue trauma involve avoiding the elevation of mucoperiosteal flaps and using piezoelectric blades and screws rather than surgical burs and chisels.

We made small holes along the distal and buccal bone of the canine teeth overlying soft tissue with tiny lesions using an orthodontic mini screw, a procedure known as “flapless osteopuncture” (OP). The trauma to the bone induces regional acceleratory phenomenon (RAP), in which the surrounding bone density decreases early on after injury, leading to a transient osteopenia (7) and thus accelerated tooth movement. The fact that the RAP maintains its activity for a certain period indicates a need to repeat the procedure.

The purpose of this study was first, to evaluate the effects of flapless osteopunctures on OTM in both the maxilla and mandible, and second, to investigate whether repeating the procedure monthly might further trigger the RAP and thus accelerate the tooth movement.

## Material and Methods

-Clinical Study

This randomized, single-center, single-blinded study was approved by the ethical committee of -------. A total of 36 patients [18 in the experimental group (OP) and 18 in the control group] requiring the removal of first premolar teeth were included in the study. The mean age was 15.27±1.62 years for the OP group and 16.13±1.28 years for the control group. All patients had to fulfill the following criteria: 1- healthy systemic condition, 2- completion of the pubertal period, 3- no medication, and 4- no previous orthodontic treatment. For each patient in the OP group, a similar patient in terms of age, malocclusion type, and anchorage need was included in the control group. An informed consent was obtained from all individual participants included in the study. One patient in the OP group and three in the control group were excluded from the study because of various reasons ( Table 1). A single examiner administering the treatment was aware of the inclusion of subjects into groups and was therefore not blinded.

**Table 1:**
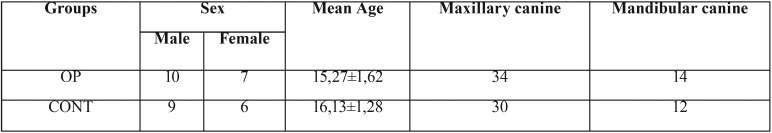
Characteristics of patients and numbers of teeth included.

**Figure 1:**
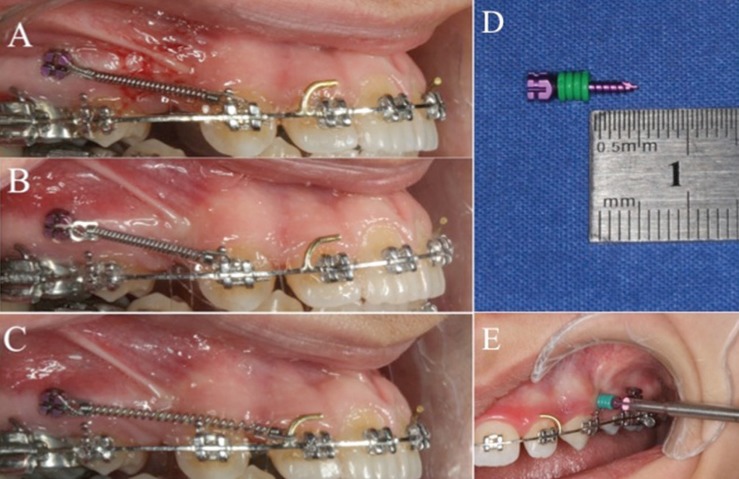
Intraoral views of canine distalization progress (A-B-C) A. İmmediately after (T1) OP creation B. First month (T3) of canine distalization (OPs were repeated) C. Second month (T4) of canine distalization (end of the study) D. Modified miniscrew for OP creation E. Clinical application of OPs.

Extractions were performed after initial records (T0) were taken from the patients. Treatment was initiated by bonding the fixed appliances (0.022” McLaughin, Bennett, and Trevisi –MBT- prescription) in both arches so that 10 days after tooth extraction were not exceeded. Passive lacebacks were performed, and transpalatal arches were placed in the maxilla. Both groups were then leveled and aligned [0.014” heat-activated NiTi (HANT; 3M Unitek, Monrovia, CA, USA); 0.016” HANT; and 0.019”–0.025” HANT]. Distalization was performed on passive 0.019”–0,025” brass-posted archwires. A mini-screw (MTN-2, DesignMed, İstanbul, Turkey) with a diameter of 1.6 mm and a length of 8 mm was modified by placing four elastomeric ligatures on the body of the mini-screw (Fig. 1). The required perforation depth was set to 5 mm. Subsequently, the mini-screws were sterilized with elastomeric ligatures and then prepared for the surgical procedure.

Infiltration anesthesia (0.5 ml Ultracain DS Forte) was applied in the OP group. Three OPs were performed in the distal aspects of the canine teeth as close to the apical as possible to achieve more bodily movement and were aligned vertically toward the attached gingiva (Fig. 1). Care was taken to ensure a distance of one mini-screw diameter between each perforation. In both groups, mini-screws were placed between the roots of the second premolar and those of the first molar teeth to support anchorage. A force of 150 g was applied by ligating the calibrated NiTi closing coil spring (Sentalloy, 3M Unitek, USA) between the orthodontic mini-screw and the canine bracket hook with a 0.010” ligature wire for canine distalization.

Subsequently, the OP procedure was repeated in the fourth week of distalization in the experimental group. Alginate impressions and photographs were taken at the beginning of the study (T0) and at the beginning (T1), first (T2), fourth (T3), and eighth week (T4) of distalization ( Table 2 and Fig. 1D). The study was concluded in the eighth week of distalization.

**Table 2:**
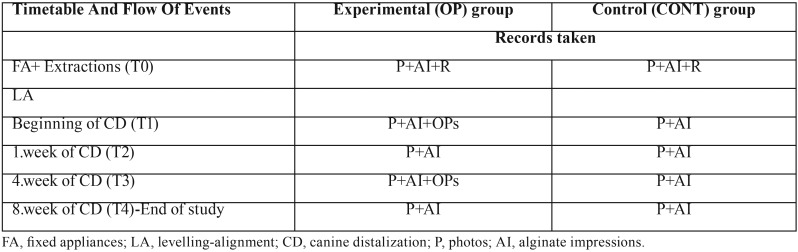
Timetable and flow of events during the clinical study.

-Measurements

The models were scanned and digitized by a software program (Orthoanalyzer, 3Shape, Copenhagen, Denmark) and superimposed at time intervals of T1–T2, T1–T3, and T1–T4, respectively. The software program was used to measure canine distalization (CD), canine rotation (CR), and canine tipping (CT) and molar mesialization (MM). A single examiner responsible for the digital measurements and data analysis was blinded. Intra-examiner reliability was determined by repeating each measurement twice at least 2 weeks apart. Correlation analysis gave r = 0,971 for angular and r = 0,986 for linear measurements. Random errors were calculated and found not to exceed ± 0.4° for angular measurements and ± 0.1 mm for linear measurements.

-CD

In measuring CD, a plane formed by the contact point of the central incisor teeth and the mesiopalatinal cusps of the first molar teeth was determined in the superimposed model. On the plane, the cusps of the canine teeth on the same side were marked, and the distance between the two cusps was measured and recorded. Fischer (8) and Aksakallı *et al.* (9) also used cusp tips in their studies.

-MM

MM was measured in a similar manner to canine by using the mesiopalatinal cusps of the first molar teeth.

-CT

In measuring CT, the angle formed between the line through the cusp tip and the deepest gingival point of the canine crown in the superimposed model was defined as the distal tipping angle. Kiliaridis *et al.* (10) also used this method to measure the first molar tipping.

-CR

**Figure 2:**
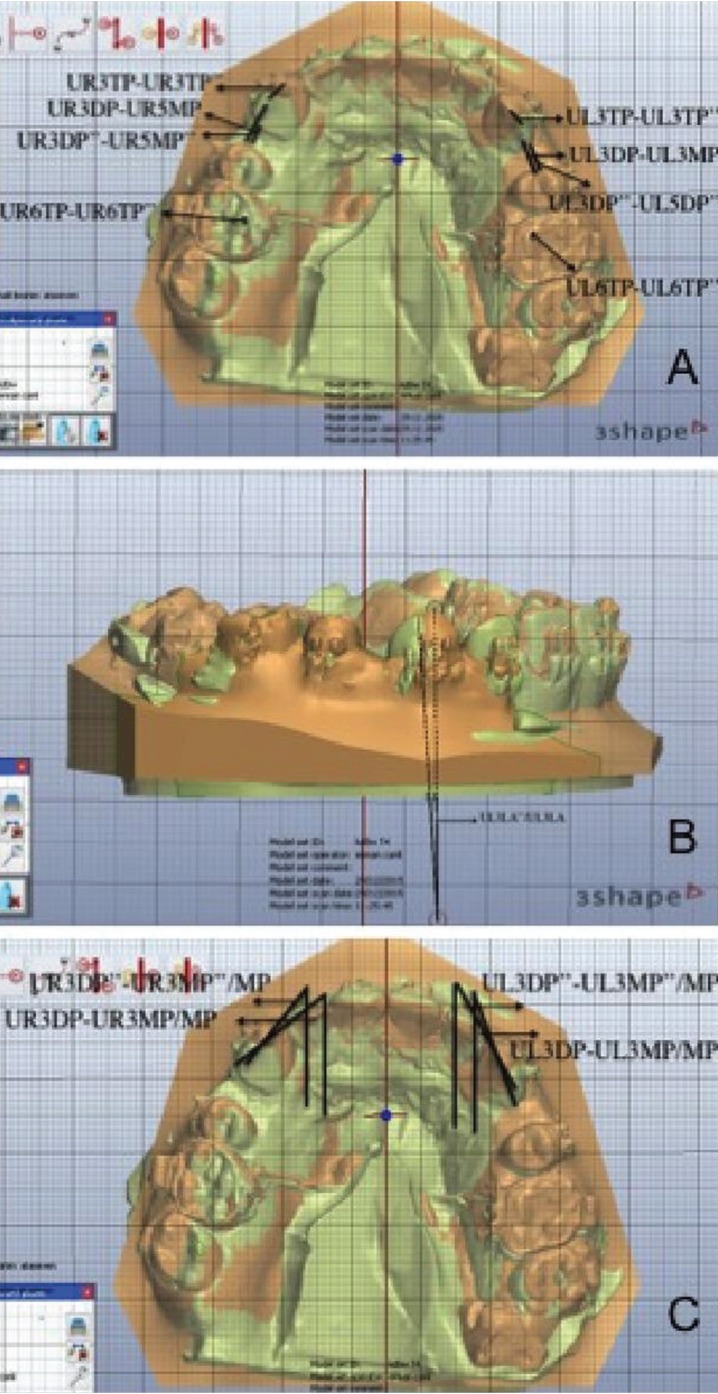
Measurement of canine distalization (A), canine tipping (B) and canine rotation (C) on the superimposed digital models.

Rotational change was measured using the digital superimposed casts with the method of Ziegler and Ingervall (11). The angle formed between the midpalatal raphe and the line through the mesial and distal contact points of the canine was defined as the distopalatinal rotation angle and was measured (Fig. 2).

-Statistical analysis

Independent Student’s t-test was used for the statistical analysis of the differences between the groups with normal distribution. For non-normal distribution data, intragroup comparisons were performed with the Wilcoxon signed-rank and intergroup comparisons with the Mann–Whitney U-test. All tests were performed at 95% (*p* = 0.05), 99% (*p* = 0.01), and 99.9% confidence interval (*p* = 0.001). The SPSS for Windows statistical package version 23.0 (SPSS Inc., Chicago, IL) was used for all descriptive statistics and analyses. The level of significance was set to *P*<.05.,

## Results

Although statistically significant differences were found between the lower and upper jaws in both groups, no difference was found between the right and left sides. For this reason, the lower and upper jaws were evaluated separately, but the right–left data of the same jaw were pooled. For all subsequent statistical analyses, the unified version of the right–left data of the same jaw was used.

The data on both OP and control groups were evaluated under two main headings: intragroup and intergroup.

-Intragroup comparison

Only the CD rate was examined and compared between the first four weeks (T1–T3) and the second four weeks (T3–T4) of distalization both for the OP and control groups. In the OP group, the CD rate was statistically significantly higher in the first four weeks (1.76 mm) than in the second four weeks (0.83 mm) only in the maxilla (*p*<0.05). In the control group, no statistically significant differences were found for the CD rate between the first four weeks and the second four weeks both in the maxilla and in the mandible.

-Intergroup comparison

The four parameters (CD, CR, CT, and MM) were assessed in the intergroup comparison. In the OP group, the upper CD rate was 0.76 mm at T2 and 1.76 mm at T3. These values were significantly higher than those in the control group. The upper canine teeth showed a statistically significant rate of tipping at T3 and T4. For the maxilla, CR and MM were not significantly different from those in the control group at any of the time intervals. Moreover, no statistically significant differences were found in the mandible for all the parameters ( Table 3).

**Table 3:**
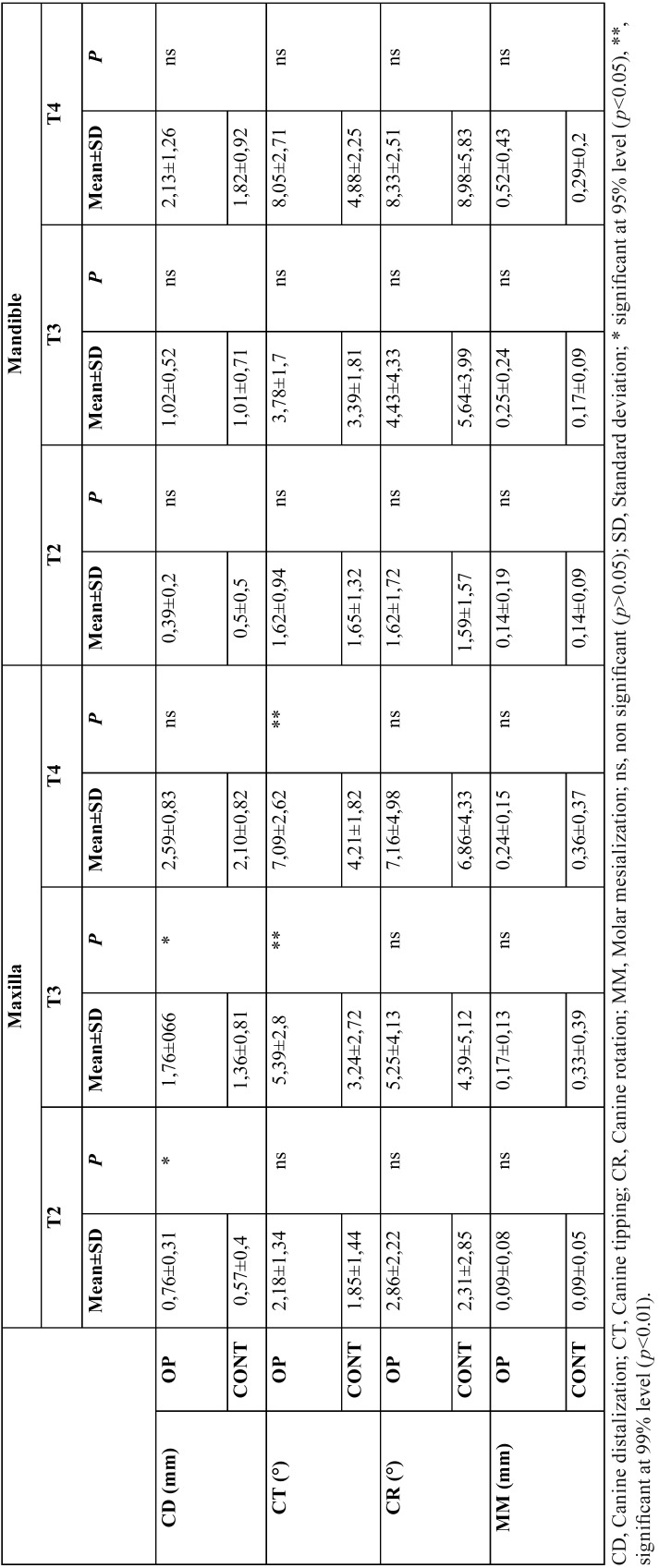
Results of the study.

## Discussion

Canine retraction with conventional methods is one of the most time-consuming parts of treatment. The monthly rate of OTM in cases treated with traditional methods when a continuous force is applied is 0.8–1.2 mm (12). Tsai *et al.* (13) reported an acceleration of 1.54 times in the micro-osteoperforation group and 1.49 times in the corticision group. Baloul *et al.* (14) reported that tooth movement was 1.3 times faster on day 42 after 10 micro-osteoperforations with flap elevation in rats. Kim *et al.* (15) created cortical punctures into the bone, a method named “piezopuncture.” These researchers reported an acceleration rate as compared with conventional mechanics of 3.26-fold in the maxilla and 2.45-fold in the mandible. Alikhani *et al.* (6) showed that the application of micro-osteoperforations at a 2–3 mm depth could increase the rate of CD by more than two-fold in the maxilla.

**Figure 3:**
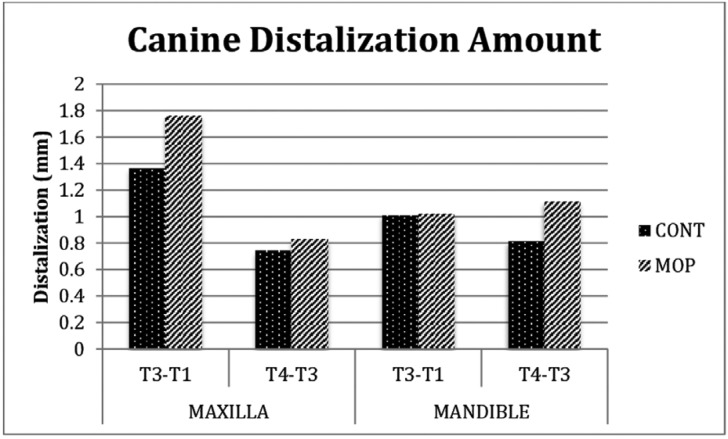
Canine distalization amount between time intervals (intragroup differences).

In the current study, the CD rate was found to be higher at T1–T3 than at T3–T4 in the maxilla, while, conversely, it was found to be higher at T3–T4 than at T1–T3 in the mandible ( Table 3 and Fig. 3). The difference in the maxilla was found to be statistically significant. In the OP (experimental) group, the CD rate in the maxilla was greater than that in the control group at T2 and T3, and no statistically significant differences were found in the mandible at any of the time intervals. Both intragroup and intergroup results were consistent with the findings of Deguchi (16) and Kim (15), who reported that tooth movement in the mandible occurred less and later than that in the maxilla. It appears that OPs do not disrupt this finding. The present results also revealed that more and faster tooth movement occurred in the maxilla at the time interval of T1–T3 with OPs than without OPs, which was similar to the findings of many studies evaluating the effect of minimally invasive surgery techniques (9,15,17). Moreover, the greatest acceleration (0.76 mm) occurred at T2, which was the first week of the OP application. This acceleration could have been due to the effect of OPs damaging the spongiosis structure of the maxilla.

In our study, CR and MM were not found to be statistically significant between the groups for either arch. This result is consistent with those of other studies suggesting that accelerating tooth movement does not lead to significant MM and CR (17,18) Alikhani *et al.* (6) revealed some tipping despite the use of power arms to make the canine teeth move bodily. Similarly, the upper CT was found to be statistically significant at T3 and T4. Thus, the OP technique can be considered to lead to more tipping in the upper canine teeth.

In the literature, information regarding how long the RAP effect lasts after trauma is applied varies; this is due to lack of standardized protocols, such as decortication shape, depth, and mucoperiostal flap reflection (4,13,18). In the current study, the mean perforation depth was determined on the basis that the perforations to be performed at depths of less than 5 mm could not cross over the gingival thickness through the medullary bone. Alikhani *et al.* (6) reported that all the inflammatory markers except IL-1 decreased to the pre-retraction levels by the 28th day of retraction. Considering the decreased effectiveness of the surgical insult on the fourth week of retraction (and, therefore, the level of cytokines and RAP response), we replaced the OPs at T3. However, our reapplication of OPs at the end of the first four weeks did not positively contribute to the CD rate for either jaw.

In this study, we created OPs by using mini-screws, because they have the advantages of wide usage, ease of application, and patient acceptance (18,19).

Although radiographs and plaster models have been reported to measure the angular changes in the canine teeth (10,20), no studies using digital models have been found. In our study, the measurement intervals of the canine tooth movement were too frequent to take an X-ray film, and doing so, would not have been ethical. Thus, angular measurements of the canine teeth were made in digital models using research conducted with plaster models and radiographs (11,21) as a guide.

Nickel-titanium closing-coil springs with an applied force of 150 g were used. Researchers have applied a similar amount of force (100–200 g) in studies in which surgical acceleration techniques were used (6,8,9,20).

The third palatal rugae have been shown to be a stable reference point for the superimposition of the maxilla (11,22), so this point was used here, also. There is currently no evidence for a clear and stable reference point for the superimposition of the mandible in the literature. In our study, distalization was performed on a 0.019”–0.025” brass-posted archwire in a 0.022” slot. Given that the friction on this size of archwire is sufficient to minimize the lower central incisor and the second molar movement during CD, these teeth were chosen as the reference points.

No harmless effects or complications related to the OPs were observed in this study. As OP is a promising and safe method due to its noninvasive nature, this study aimed to evaluate its effects on the CD rate by testing a new application protocol and frequency in both the maxilla and the mandible. Further OP application protocols should be performed to examine the ideal timing to induce RAP in order to trigger the tooth movement rate.

## Conclusions

1. OPs, as applied in this study, significantly increased the CD rate at the T1–T3 time interval in the maxilla.

2. The recreation of OPs at T3 did not trigger the CD rate;

3. Tooth movement with OPs occurred significantly faster in the maxilla than in the mandible.

## References

[1] Huang H, Williams RC, Kyrkanides S (2014). Accelerated orthodontic tooth movement: molecular mechanisms. Am J Orthod Dentofacial Orthop.

[2] Kole H (1959). Surgical operations on the alveolar ridge to correct occlusal abnormalities. Oral Surg Oral Med Oral Pathol.

[3] Wilcko WM, Wilcko T, Bouquot JE, Ferguson DJ (2001). Rapid orthodontics with alveolar reshaping: two case reports of decrowding. Int J Periodontics Restorative Dent.

[4] Yi J, Xiao J, Li Y, Li X, Zhao Z (2017). Efficacy of piezocision on accelerating orthodontic tooth movement: A systematic review. Angle Orthod.

[5] Kim SJ, Park YG, Kang SG (2009). Effects of Corticision on paradental remodeling in orthodontic tooth movement. Angle Orthod.

[6] Alikhani M, Raptis M, Zoldan B, Sangsuwon C, Lee YB, Alyami B (2013). , Effect of micro-osteoperforations on the rate of tooth movement. Am J Orthod Dentofacial Orthop.

[7] Verna C (2016). Regional Acceleratory Phenomenon. Front Oral Biol.

[8] Fischer TJ (2007). Orthodontic treatment acceleration with corticotomy-assisted exposure of palatally impacted canines. Angle Orthod.

[9] Aksakalli S, Calik B, Kara B, Ezirganli S (2016). Accelerated tooth movement with piezocision and its periodontal-transversal effects in patients with Class II malocclusion. Angle Orthod.

[10] Kiliaridis S, Lyka I, Friede H, Carlsson GE, Ahlqwist M (2000). , Vertical position, rotation, and tipping of molars without antagonists. Int J Prosthodont.

[11] Ziegler P, Ingervall B (1989). A clinical study of maxillary canine retraction with a retraction spring and with sliding mechanics. Am J Orthod Dentofacial Orthop.

[12] Andrade I Jr, Sousa AB, da Silva GG (2014). New therapeutic modalities to modulate orthodontic tooth movement. Dental Press J Orthod.

[13] Tsai CY, Yang TK, Hsieh HY, Yang LY (2016). Comparison of the effects of micro-osteoperforation and corticision on the rate of orthodontic tooth movement in rats. Angle Orthod.

[14] Baloul SS, Gerstenfeld LC, Morgan EF, Carvalho RS, Van Dyke TE, Kantarci A (2011). Mechanism of action and morphologic changes in the alveolar bone in response to selective alveolar decortication-facilitated tooth movement. Am J Orthod Dentofacial Orthop.

[15] Kim YS, Kim SJ, Yoon HJ, Lee PJ, Moon W, Park YG (2013). Effect of piezopuncture on tooth movement and bone remodeling in dogs. Am J Orthod Dentofacial Orthop.

[16] Deguchi T, Takano-Yamamoto T, Yabuuchi T, Ando R, Roberts WE, Garetto LP (2008). Histomorphometric evaluation of alveolar bone turnover between the maxilla and the mandible during experimental tooth movement in dogs. Am J Orthod Dentofacial Orthop.

[17] Aboul-Ela SM, El-Beialy AR, El-Sayed KM, Selim EM, El-Mangoury NH, Mostafa YA (2011). Miniscrew implant-supported maxillary canine retraction with and without corticotomy-facilitated orthodontics. Am J Orthod Dentofacial Orthop.

[18] Sayin S, Bengi AO, Gurton AU, Ortakoglu K (2004). Rapid canine distalization using distraction of the periodontal ligament: a preliminary clinical validation of the original technique. Angle Orthod.

[19] Shpack N, Davidovitch M, Sarne O, Panayi N, Vardimon OP (2008). Duration and anchorage management of canine retraction with bodily versus tipping mechanics. Angle Orthod.

[20] Davidovitch Z, Finkelson MD, Steigman S, Shanfeld JL, Montgomery PC, Korostoff E (1980). Electric currents, bone remodeling, and orthodontic tooth movement. II. Increase in rate of tooth movement and periodontal cyclic nucleotide levels by combined force and electric current. Am J Orthod.

[21] Almeida MA, Phillips C, Kula K, Tulloch C (1995). Stability of the palatal rugae as landmarks for analysis of dental casts. Angle Orthod.

[22] Hoggan BR, Sadowsky C (2001). The use of palatal rugae for the assessment of anteroposterior tooth movements. Am J Orthod Dentofacial Orthop.

